# Fabrication and Packaging of CMUT Using Low Temperature Co-Fired Ceramic

**DOI:** 10.3390/mi9110553

**Published:** 2018-10-27

**Authors:** Fikret Yildiz, Tadao Matsunaga, Yoichi Haga

**Affiliations:** 1 Graduate School of Engineering, Tohoku University, 6-6 Aza-Aoba, Aramaki Aoba-ku, Sendai 980-8579, Japan; 2 Faculty of Engineering, Hakkari University, Hakkari 30000, Turkey; 3 Graduate School of Biomedical Engineering, Tohoku University, 6-6 Aza-Aoba, Aramaki Aoba-ku, Sendai 980-8579, Japan; matsunaga@tohoku.ac.jp (T.M.); haga@tohoku.ac.jp (Y.H.)

**Keywords:** capacitive micromachined ultrasonic transducers (CMUT), low temperature co-fired ceramic (LTCC), LTCC side via, indirect packaging

## Abstract

This paper presents fabrication and packaging of a capacitive micromachined ultrasonic transducer (CMUT) using anodically bondable low temperature co-fired ceramic (LTCC). Anodic bonding of LTCC with Au vias-silicon on insulator (SOI) has been used to fabricate CMUTs with different membrane radii, 24 µm, 25 µm, 36 µm, 40 µm and 60 µm. Bottom electrodes were directly patterned on remained vias after wet etching of LTCC vias. CMUT cavities and Au bumps were micromachined on the Si part of the SOI wafer. This high conductive Si was also used as top electrode. Electrical connections between the top and bottom of the CMUT were achieved by Au-Au bonding of wet etched LTCC vias and bumps during anodic bonding. Three key parameters, infrared images, complex admittance plots, and static membrane displacement, were used to evaluate bonding success. CMUTs with a membrane thickness of 2.6 µm were fabricated for experimental analyses. A novel CMUT-IC packaging process has been described following the fabrication process. This process enables indirect packaging of the CMUT and integrated circuit (IC) using a lateral side via of LTCC. Lateral side vias were obtained by micromachining of fabricated CMUTs and used to drive CMUTs elements. Connection electrodes are patterned on LTCC side via and a catheter was assembled at the backside of the CMUT. The IC was mounted on the bonding pad on the catheter by a flip-chip bonding process. Bonding performance was evaluated by measurement of bond resistance between pads on the IC and catheter. This study demonstrates that the LTCC and LTCC side vias scheme can be a potential approach for high density CMUT array fabrication and indirect integration of CMUT-IC for miniature size packaging, which eliminates problems related with direct integration.

## 1. Introduction

The capacitive micromachined ultrasonic transducer (CMUT) is an advanced ultrasonic transducers technology and is based on a micro electro mechanical systems (MEMS). The simple structure of CMUT consists of a micromachined membrane suspended over a cavity, a fixed bottom electrode, and a top electrode [1,2]. It has attracted scientists and researchers in this field in recent years. There are several studies related to numerical and analytical methods of CMUT in addition to fabrication [3,4,5,6,7,8,9]. First generation CMUTs were fabricated using the surface micromachining/sacrificial layer releasing method [10]. This method includes several depositions and etching steps. Cavities under the membrane are obtained by selective etching of the sacrificial layer through etching holes that are patterned on the membrane and this reduces, however, the active area of membrane (fill factor). The membrane is deposited over the sacrificial layer and an additional deposition step is required to seal cavities. Each of the deposition steps induces stress on the membrane [11,12,13,14,15,16]. Thus, precise control over membrane thickness is very critical because it determines the mechanical properties of the membrane (low internal stress, mechanical loss etc.). Moreover, the other common problem of sacrificial layer releasing for cavity formation is stiction which occurs after selective etching of the sacrificial layer. Capillary forces on the membrane during drying of water in the cavity push the membrane to the bottom substrate and break if the membrane is not sufficiently thick [17].

Wafer bonding was introduced as an alternative to surface micromachining and provides simplicity, flexibility, and superior control over fabrication processes and material selections [17]. In wafer bonding, a single silicon crystal is used as a membrane and vacuum sealed cavities are achieved without opening etch holes on the membrane, both of which directly translate into a high performance device with high fill factor [18]. Fusion bonding and anodic bonding are mostly preferred wafer bonding methods for 1D/2D CMUT fabrication among other bonding techniques due to the advantages of bond strength, reliability, and hermiticity [19,20,21,22,23,24]. However, high bonding temperature, and the flat and clean bonding surface requirement are limitations of fusion bonding [25]. Anodic bonding, on the other hand, is a promising candidate for CMUTs fabrication and packaging (electronic integration) due to low temperature process compatibility. A CMUT uses all the benefits of advanced MEMS technology; however, it still needs improvements to show comparable performance to its piezoelectric counterpart in terms of sensitivity and output pressure. Due to small capacitance, CMUTs are sensitive to parasitic capacitance and have a low SNR (signal to noise ratio) value [26,27,28]. Low output pressure is other concern with CMUT performance. For ultrasound imaging and therapeutic applications, high SNR and output pressure are the main requirements as well as high dynamic range and low cross coupling between transducer elements [29,30,31,32]. To do this, the active area of the vibrating membrane would be increased and parasitic effects should be minimized. Direct integration of CMUT and front-end electronics (3D integration) is highly desirable to increase SNR and output pressure, but also reduction of parasitic effects. Thus, through-wafer interconnects are needed and electrical contact pads have to be located at the backside of the CMUT for 3D packaging and to provide communication between CMUT elements and the IC chip. Several materials and methods have been under investigation to show CMUT packaging with electronics. Earlier through-wafer interconnects efforts were widely through silicon via (TSV) [33]. The TSV process begins with vias opening on silicon substrate by deep reactive ion etching (DRIE) and then thermal oxidation of substrate for insulation. The next step is filling vias with a conductive material such as polysilicon which serves as conductor between the front side (CMUT) and backside (bonding pad) of the wafer. These TSV processes induce stress on the silicon substrate and require an additional polishing step to achieve a bondable surface for fusion bonding [34]. Alternatively, the through trench isolation approach has been announced to eliminate drawbacks related with the TSV method [35]. For example, a process has recently been reported for the fabrication of a CMUT array with isolation trenches using anodic bonding [36]. This study proposed a simple interconnects formation without through-wafer via. To date, the majority of works have focused on through-silicon vias (TSV), however, parasitic capacitance is an issue for such architecture. Using dielectric material in the form of through-glass vias (TGV) rather than Si can eliminate these undesired effects and low surface roughness is not needed for bonding [37,38]. Via formation and metallization of glass are not an easy and simple task although promising results of CMUT fabrication using Through Glass via (TGV) have been shown [39]. An alternative material called anodically bondable low temperature co-fired ceramic (LTCC) has been developed, which has been widely used for die level or wafer level MEMS packaging over past years. A narrower via pitch fabrication is easier than when using a glass substrate, and also LTCC allows freedom in via design [40,41,42,43]. Recently, SOI-LTCC anodic bonding has been announced for CMUT fabrication [24,44,45]. In these studies, CMUTs were built directly on open tool and customized LTCC substrate. Fabricated devices were electromechanically characterized for resonance frequency in air and immersion medium. Initial results showed that LTCC is one of the potential candidates for CMUT fabrication and hybrid integration with electronics. Moreover, LTCC has via and vertical interconnects which enables lateral side via architecture (indirect packaging) for electronic integration with IC. This is highly desirable for small size CMUT packaging, for example, tube shaped packaging of CMUT to visualize the narrower part of the vessel (intravascular imaging). In other words, lateral side and backside integration of CMUT with electronics are possible with LTCC substrate [46]. All aforementioned advantages of LTCC might provide high-density CMUT array fabrication and 3D packaging for different applications.

In this study, a custom designed LTCC wafer was used for CMUT fabrication and packaging. Bottom electrodes were directly built on LTCC via and high conductive silicon was used as top electrode and cavity formation. Anodic bonding of LTCC-SOI substrate was the final step of the fabrication process. A novel packaging process was introduced by using lateral side vias of LTCC that were achieved by micromachining of fabricated CMUT device. This packaging process refers to indirect integration of CMUT and IC using an intermediate material (catheter). Hexagonal shaped CMUTs with lateral side via were assembled with a catheter. ICs were mounted on the catheter following patterning of connection and contact pads using flip-chip bonding. Flip-chip bonding performances were evaluated and compared with similar studies in literature. Finally, the pros and cons of fabrication and packaging of LTCC based CMUTs were evaluated and discussed.

## 2. Materials and Methods

### 2.1. Fabrication

**Figure 1:**
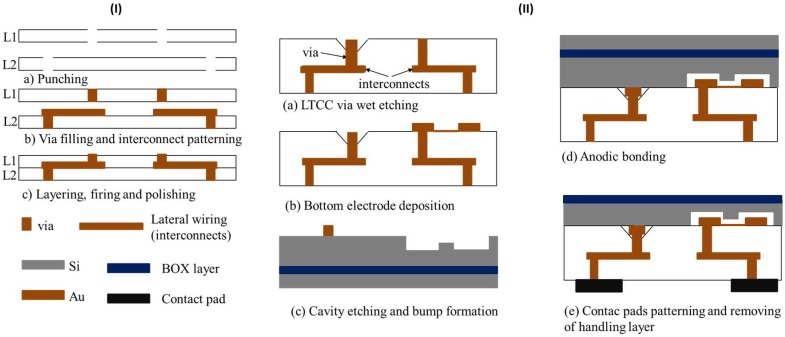
(**I**) Low temperature co-fired ceramic (LTCC) fabrication: (**a**) Via hole opening by punching; (**b**) Filling hole and interconnects patterning; (**c**) Layering and firing. (**II**) Capacitive micromachined ultrasonic transducer (CMUT) fabrication process: (**a**) Porous via formation by LTCC wet etching; (**b**)Bottom electrode deposition; (**c**) Cavity etching and Au-bump deposition; (**d**) Anodic bonding and (**e**) Contact pad patterning and handling layer removing.

**Figure 2:**
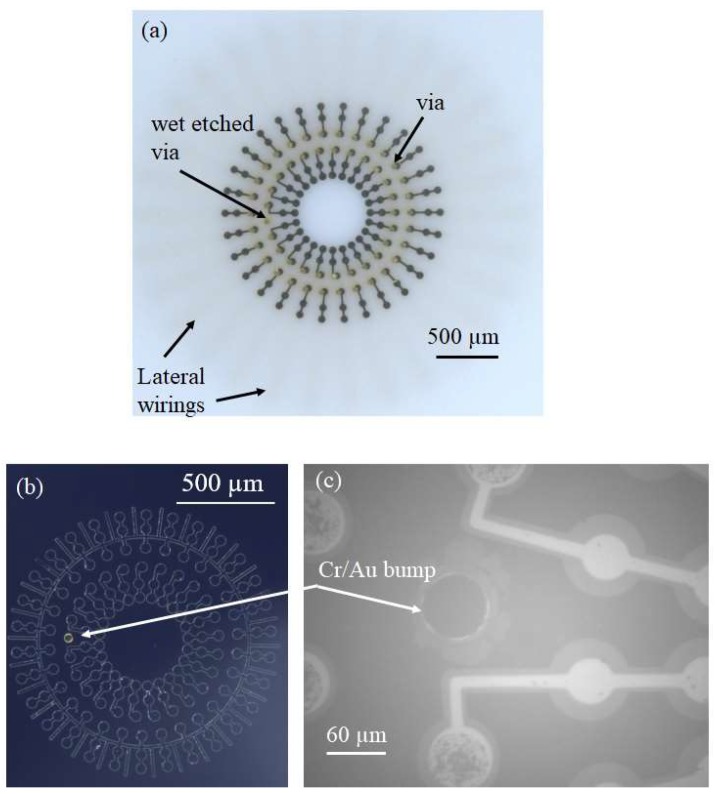
Preparation of LTCC and Si substrate for anodic bonding (Top view). (**a**) Bottom electrodes on dual ring LTCC with a number of 25 inners and 30 outer via; (**b**) Bumps and cavities on Si and (**c**) IR view of device patterns of CMUT after handling layer removing.

LTCC is a substrate made of a mixture of ceramic powder known as green sheet and a glass powder. Via fabrication is based on the following steps: (1) punching of green sheet for via hole formation, (2) screen printing of vias and interconnects (lateral wiring), and (3) stacking and firing of green sheet, respectively [42,43], as illustrated in Figure 1-(I). LTCC used in this study consists of vias with a diameter of 60 µm. However, LTCC has a 30 µm fabrication error. Lateral wiring (interconnects) and vias are made of conductive materials (Au) and provide electrical connections between the top and bottom of the LTCC substrate. LTCC and SOI substrates with a size of 2 cm × 2 cm were used for CMUT fabrication. The CMUT fabrication process was briefly summarized in Figure 1-(II). Table 1 shows CMUT fabrication parameters. In this micromachining process, the SOI wafer was firstly cleaned with Piranha solution (H_2_SO_4_:H_2_O_2_ = 2:1) to remove organics and contaminants. A thin layer of Au/Cr (20/30 nm) was sputtered on both sides of the LTCC wafer as a mask layer for wet etchant, and positive photoresist (PMER P-LA900, Tokyo Ohka Kogyo Co., Ltd., Kanagawa, Japan) with a thickness of 8 µm was spin coated for the lithography process. LTCC substrate was then exposed to wet etchant (HF:H_2_SO_4_ = 85:15) to obtained porous LTCC via. For 30 s etching time, etching depth and diameter of LTCC via were 10 µm and 150 µm, respectively. Bottom electrodes made of Au/Pt/Cr (70/30/20 nm) were patterned on the remaining LTCC via by a lift-off process after removal of resist and metals. Positive photoresist of 2 μm thick (OFPR-800 LB 200 cP, Tokyo Ohka Kogyo Co.,Ltd, Kanagawa, Japan) was used for photolithography and the lift-off process of the bottom electrode formation (Figure 2a). In the view of the SOI substrate, 0.4 µm circular shape cavities were micromachined on the high conductive Si part of SOI by reactive ion etching (RIE) with SF6 gas and Si used as a top electrode. Cr/Au metal bump with a thickness of 1.2 µm were sputtered on Si substrate for bonding with wet etched LTCC via during anodic bonding (Figure 2b). Anodic bonding of LTCC-SOI substrates was completed under a high vacuum condition at 380 °C. We repeated anodic bonding 27 times, resulting in 243 single CMUTs die. Because 2 cm × 2 cm LTCC includes nine different via designs comprising a linear and circular array. Au-Au bonding of wet etched LTCC vias and bumps forms an electrical connection between the top and bottom of the bonded sample [40,41]. The undesired part of bonded LTCC-SOI substrates (handling layer-300 µm in thickness) was then removed by deep reactive ion etching (DRIE) following by contact pads formation at the backside of LTCC (Figure 2c). Fabricated CMUTs were characterized in air and immersion medium. More details about LTCC-SOI bonding process flow have been given in our previous studies [44].

### 2.2. Packaging

**Figure 3:**
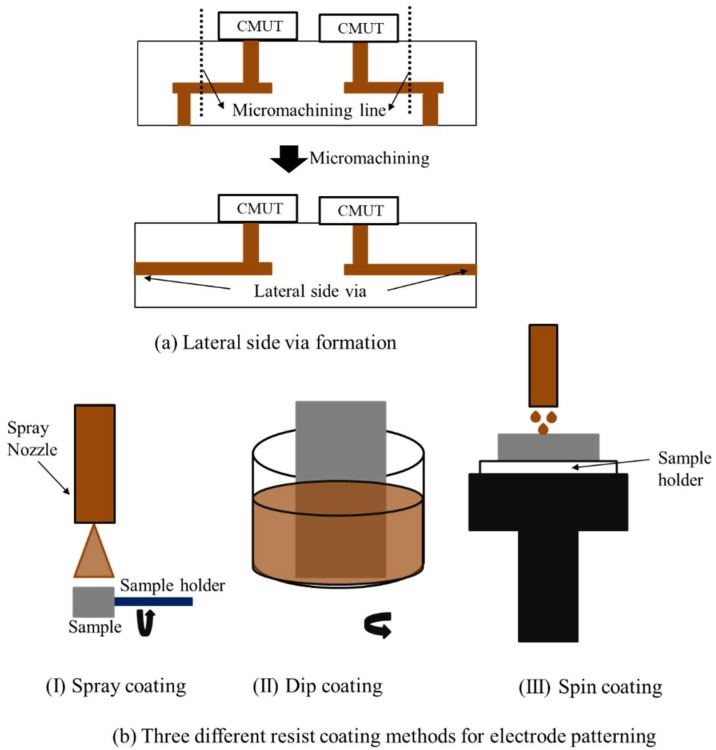
CMUT packaging process flow. (**a**) Side via formation and (**b**) electrode patterning methods.

Packaging process flow of indirect CMUT-IC packaging is described in this section of paper. This novel packaging process uses the lateral side via of LTCC rather than the backside of substrate for packaging. Initial results and more details have been found in our previous research in [45,46]. According to [46], indirect connection of CMUT with lateral side via and IC circuits was proposed through patterned electrodes on the LTCC side via and catheter. The catheter is made of a biocompatible solid polyimide substrate with a size of 3 mm × 3 mm × 20 mm. This process consists of four different steps: (1) machining of fabricated CMUT and catheter, (2) assembly of CMUT and catheter, (3) electrode and contact pad patterning on both substrate, and (4) IC mounting on catheter by flip-chip bonding. Lateral side vias were obtained by cutting the CMUT device in hexagonal (Φ: 2.4 mm) and rectangular shapes (Φ: 3 mm) using the dicing machine as illustrated in Figure 3a. Diamond blades 1 mm and 0.1 mm thick were used for micromachining of the fabricated CMUT and catheter, respectively. The micromachined catheter has three different regions: first planar surface (length: 3 mm), taper (length: 2 mm), and second planar surface (length: 10 mm). The taper was formed with a 0.05 mm dicing pitch although the other part of the catheter was diced with a 0.1 mm dicing pitch. The taper depth was the sum of IC chip thickness and contact pads thickness of both flip-chip bonded samples (IC and catheter). The assembly process follows micromachining and is the mounting of the catheter to the backside of the CMUT using epoxy adhesive. Alignment of the catheter and CMUT was achieved with a set of sample holders and a fixer. They were fabricated with a 3D printer (Agilista-3000, Keyence Co., Osaka, Japan).

The first sample holder was designed and fabricated for CMUT and the other was the catheter. Holder and fixer have alignment holes and pins for assembly process. Samples were put inside the holders and high temperature resistance adhesive (EPO-TEK^®^ 353ND, Epoxy Technology Inc., Billerica, MA, USA) was then used for assembly. Alignment of samples was achieved with alignment holes and pins and samples were strictly put together by pushing the fixer at 100 °C curing temperature for around 1 h. Three different coating methods were used for connection electrodes, wiring pads, and bonding pads patterning on side via and catheter after assembly: spray coating, dip coating, and spin coating as shown in Figure 3b. The Nonplanar exposure system which consists of a UV spot laser and a computer-controlled multiaxial stage used resist patterning for spray coating and dip coating [47,48]. However, the spin coating method was preferred to electrode patterning using a planar exposure system. Lithography process results of three coating methods showed that spin coating and the planar exposure system were the best fitted methods for electrode and contact pad patterning on assembled samples. Therefore, electrodes and contact pads were patterned by spin coating and planer exposure system using a contact mask aligner (Ma8, Suss MicroTec KK, Kanagawa, Japan). Electrodes and contact pads deposition were achieved using the following steps: 1.2 µm thick Cr/Au electrodes and alignment marks were firstly patterned on assembly by a lift-off process using positive photoresist (OFPR- 800 LB 200 cP, Tokyo Ohka Kogyo Co., Ltd., Kanagawa, Japan). These electrodes provide a connection between LTCC side via and IC bonding pads that are on the catheter. Wiring pads and bonding pads were then electroplated after patterning of positive photoresist (PMER P-LA900, Ohka Kogyo Co., Ltd., Kanagawa, Japan). Wiring pads were designed and deposited for measurement of the resistance between the flip-chip bonding pads. The widths of the bonding pads and connection electrodes have widths of 70 µm and 20 µm and pitches of 140 µm and 150 µm, respectively. It was measured that thickness of deposited electrode and pads were 5 µm. A dummy IC chip was used for flip-chip bonding. Silicon-on-insulator (SOI; 3 µm/50 nm/300 µm) substrate was preferred as a dummy IC and includes eight bonding pads. Au/Cr bonding pads (80/200 nm) were first patterned and then 50 µm thick Au bumps were formed on the Au/Cr pads using a wire bonder (7700 West Bonder, West Bond Inc., Anaheim, CA, USA). Finally, an IC chip was mounted on the second planar surface of the catheter using a flip-chip bonder (FINEPLACER^®^lambda, Finetech GmbH & Co. KG, Berlin, Germany). A bonding force of 25 N was applied for 3 min and heated to 380 °C. Flip-chip bonding results were evaluated by resistance measurement of bonding pads [46].

## 3. Results

**Figure 4:**
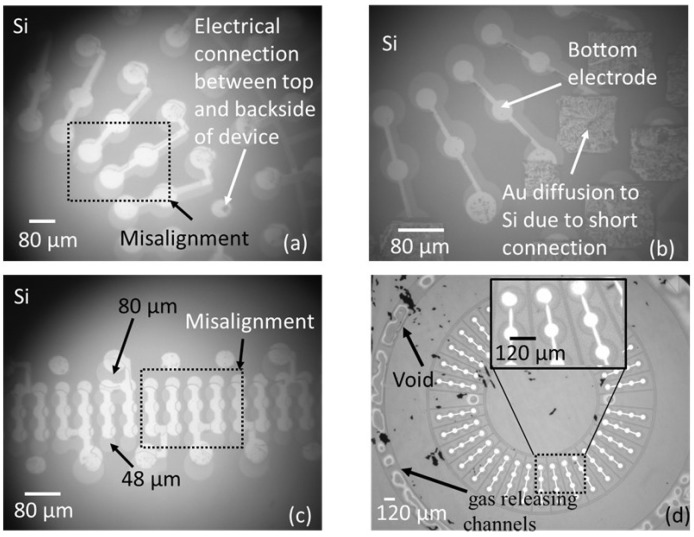
Three CMUT devices with different membrane size. (**a**) 80 µm, (**b**) Au diffusion in Si membrane, (**c**) linear array CMUT with a 48 µm membrane size, d and (**d**) CMUT ring array with a 120 µm membrane size.

Anodic bonding quality evaluation is needed to show functionality of fabricated devices. The first bond quality evaluation of the bonded sample was dicing of samples into small pieces (0.6 cm × 0.6 cm) using a dicing machine (DAD 522, DISCO Co., Tokyo, Japan). Bonding is considered as successful when bonded samples stayed together. In order to inspect the bonding quality more accurately, the fabricated devices were tested with three additional different measurements: (1) Visual inspection of bonded samples to detect misalignment using IR camera, (2) impedance analyzer for the measurement of admittance (G (conductance)-B (susceptance)) as a function of the frequency, and (3) static membrane deflection by topography measurement system (TMS) (Polytec Japan, Kanagawa, Japan) to show hermiticity of the sealed cavity. Visual inspections of bonded samples were firstly tested using the IR camera. Top views of four different CMUT devices obtained by IR camera are shown in Figure 4a,c,d. Our results showed that there was a misalignment between the top and bottom electrode of CMUTs with membrane diameter of 48 µm, 50 µm, and 80 µm. In addition to the LTCC via error (30 µm), an approximately 20 µm mechanical error was measured during samples preparation (alignment, clamping etc.) before the bonding process. It was assumed that the mechanical error related with the bonding machine was the reason for misalignment in addition to the via fabrication error. Gold diffusion into silicon was also observed due to short contact of the Si membrane and bottom electrode made of Au/Pt/Cr as a result of misalignment (Figure 4b). It was noted that the CMUT cell with a dimension of 120 µm has no misalignment as shown in Figure 4d. Misalignment and a short connection between the top and bottom electrode is also confirmed by complex admittance measurement. Conductance, G (ω), refers to the real part and susceptance, B (ω), presents the imaginary part of complex admittance. Lumped equivalent circuit and values of circuit parameters can be obtained by plotting B (ω) versus G (ω) as described in [49].

**Figure 5:**
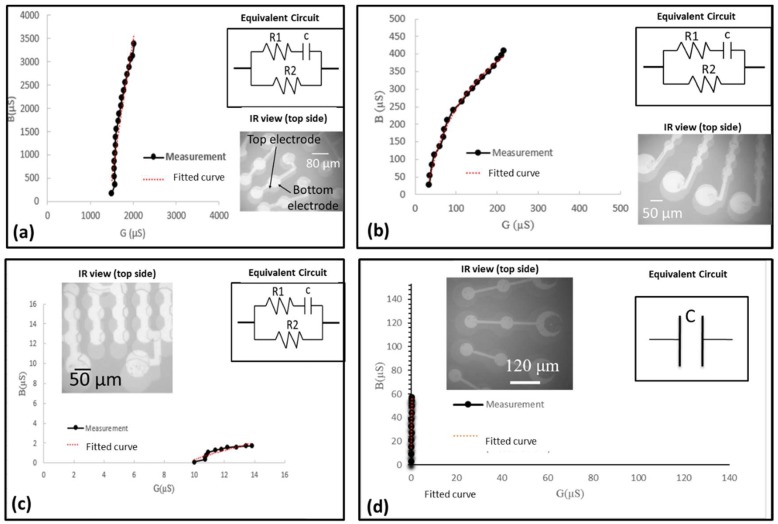
Complex admittance plot of four different CMUT devices: (**a**) 80 µm; (**b**) 50 µm; (**c**) 48 µm; and (**d**) 72 µm.

**Figure 6:**
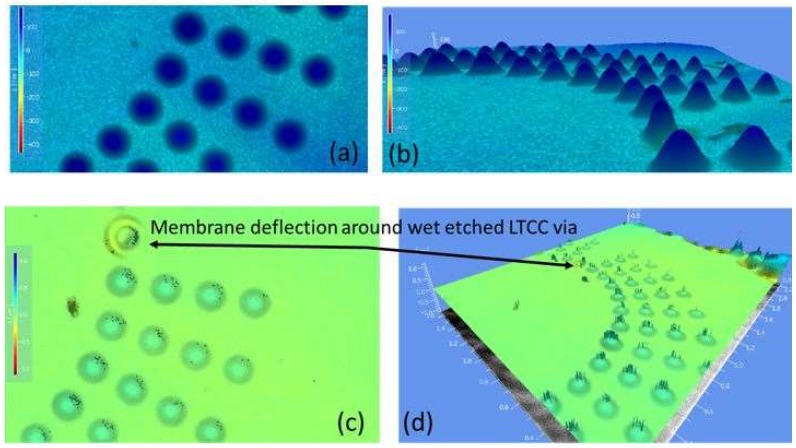
Membrane deflection of successfully bonded sample. Membrane deflection of CMUT with a 120 µm membrane size. (**a**) Top view (deflection: 162.5 nm); (**b**) 3D view (deflection: 174.5 nm). Hermitically sealed cavity around wet etched LTCC via; (**c**) Top view and (**d**) 3D view.

Complex admittance measurement by impedance analyzer (HP4194A, Hewlett Packard, Co., Palo Alto, CA, USA) was employed to obtain G (conductance)-B (susceptance) plot of fabricated devices. It simply gives an idea about the characteristics (equivalent circuit) of fabricated devices that can be a resistor, capacitor, inductor, or a combination of three electronic circuit elements. Resistance and frequency value of fabricated devices were derived by plotting the imaginary part of the admittance, B (ω), versus the real part, G (ω). G–B plot of four different CMUT designs and their equivalent circuits are shown in Figure 5 and the inset of Figure 5, respectively. Fabrication results showed that equivalent circuits of three fabricated devices (40 µm, 50 µm, and 80 µm) consist of a capacitor with a series resistor (R1) and a parallel resistor (R2). For CMUT with a 72 µm membrane size, a capacitor is the only parameter of equivalent circuits as expected. Logarithmical curve fitting was applied to find the best suited function for the first three designs, and linear curve fitting matches the data of the last design (72 µm). Lastly, TMS was used to measure static deflection of the CMUT membrane under atmospheric pressure as shown in Figure 6. It was observed that the membrane deflection profile of the Si membrane was in an upward direction. In contrast to the CMUT membrane, deflection of the Si part around the wet etched LTCC via was downward, as shown in Figure 6c,d. Mechanical and electrical characterization of CMUTs in air were determined by resonance frequency and impedance measurements. Resonance frequency of a device in air was measured using a vibrometer (UHF-120, Polytec Japan, Kanagawa, Japan), and a network analyzer (MS4630B, Anritsu, Co., Morgan Hill, CA, USA) was used for impedance measurement. CMUT with a 120 µm membrane size was used for experimentation. The measured maximum membrane displacement in air was 10.3 pm at 2.88 MHz under excitation with a 7 Vpp AC signal without DC bias voltage. A finite element model of a CMUT cell was constructed in COMSOL Multiphysics (COMSOL® version 5.2, COMSOL, Inc., Burlington, MA, USA) software, coupling the structural mechanics subdomain and the electrostatics subdomain to compare experimental and numerical results.

**Figure 7:**
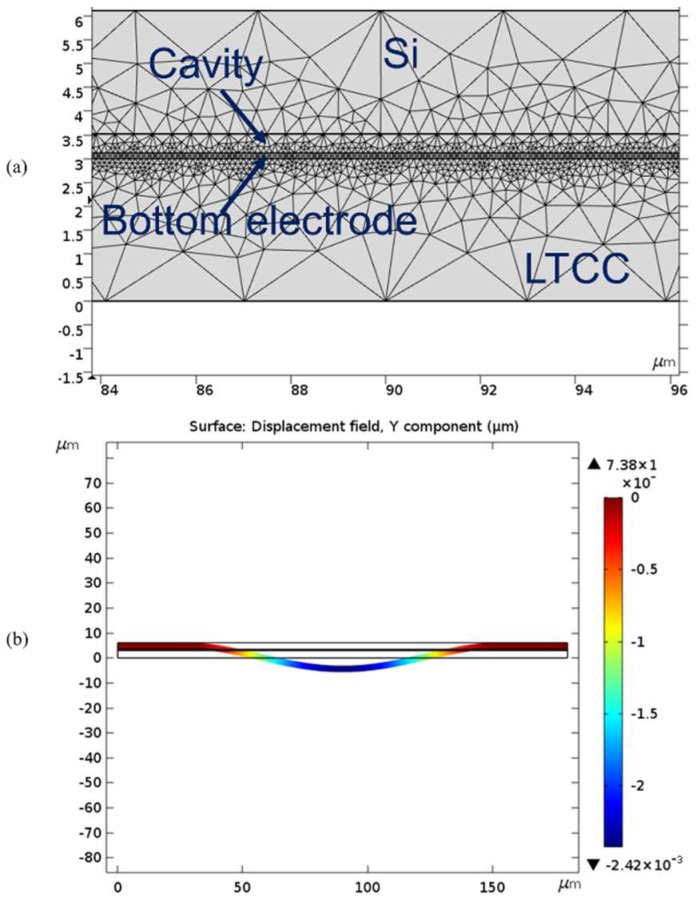
(**a**) Mesh information of 2D FEM model of a CMUT cell and (**b**) Vertical displacement under 7V DC bias.

The 2D electromechanical coupling model was used. The free triangular mesh and defaults parameters were set for calculation. The Minimum feature size was 0.054 µm. The fixed mesh was applied to LTCC and the bottom electrode when modeling. However, Si membrane and cavities were free to deform. Squeezed film damping in sealed cavity was omitted for modelling, because it was analytically proved from a previous study that the presence of air does not cause any squeeze film damping for flexural membrane [50]. Resonance frequency and maximum membrane displacement were obtained as 1.5755 MHz and 2.42 pm according to the numerical analysis as shown in Figure 7 [44].

**Figure 8:**
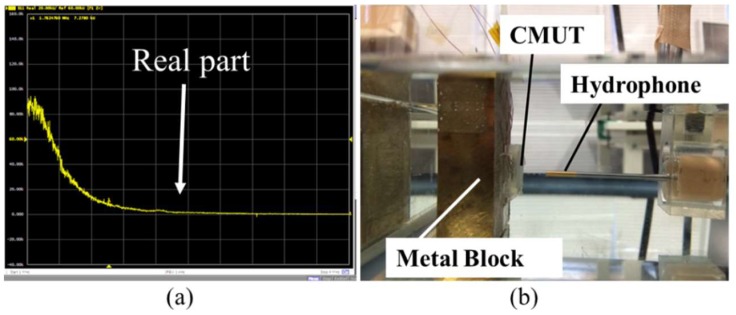
Device characterizations. (**a**) Electrical measurement by impedance analyzer and (**b**) acoustic measurement in water by hydrophone.

Experimental results of the impedance measurement are shown in Figure 8a. Applied DC voltages are changed from 10 V to 40 V and resonance span is changed from 1–4 MHz. The second experimental setup is the pitch-catch setup where one of the transducers transmits an acoustical signal, and this signal was measured by a hydrophone (TC4038, Teledyne RESON Inc., Thousand Oaks, CA, USA) placed at a distance from the CMUT surface in water as shown in Figure 8b. This hydrophone has a frequency ranging up to 20 MHz. However, we could not observe any peak around the resonance frequency. Moreover, CMUT devices used in electrical and acoustical measurement were not successfully driven by different AC and DC voltages while device fabrication was successful.

**Figure 9:**
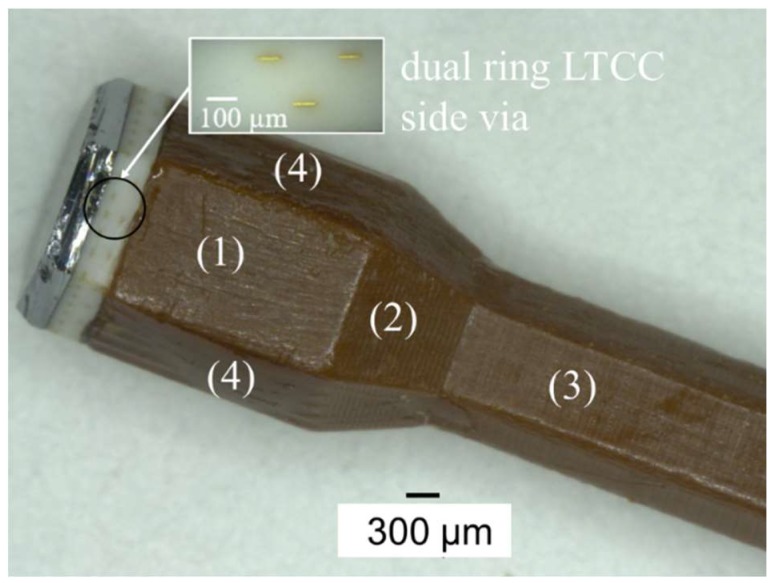
Assembled hexagonal shaped CMUT and catheter. (1) is the first planer surface of catheter; (2) taper; (3) 2nd planer surface; and (4) lateral side of catheter.

**Figure 10:**
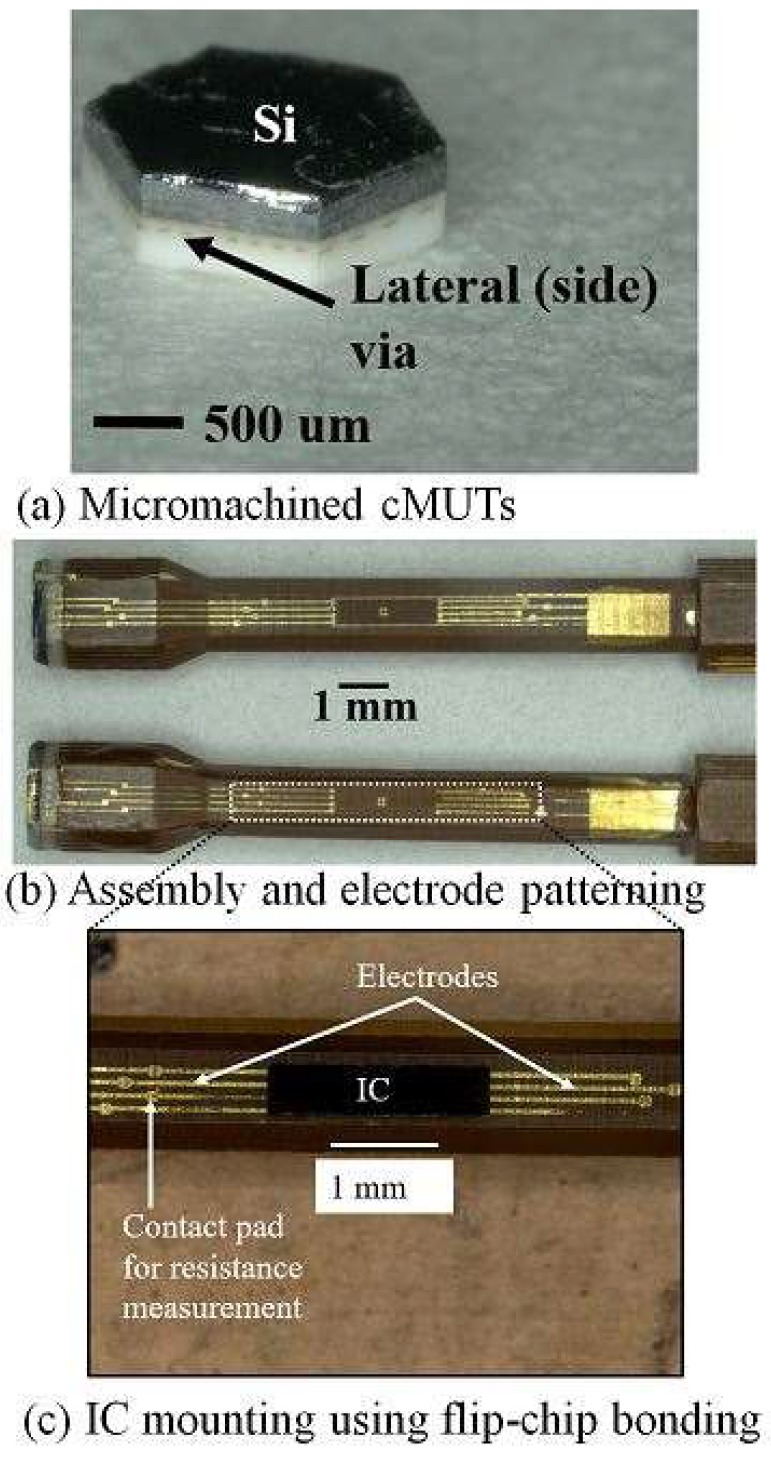
Indirect CMUT-IC packaging process using LTCC side via. (**a**) Hexagonally micromachined CMUT; (**b**) Electrodes and contact pads on catheter after mounting of catheter at the backside of CMUT and (**c**) IC mounting on catheter.

Surface roughness measurement of catheter after micromachining were the first experimental results of packaging. It was measured that the surface roughness of the lateral side of the first planar surface was about to 20–30 µm. When these surfaces were polished to reduce surface roughness, the shape of catheter turned into a circular shape. When the assembly process is considered, epoxy adhesive that is compatible with low temperatures is not a good choice for the assembly of CMUT and catheter (Figure 9). Experimental results showed that amount of adhesive should be confirmed before the assembly process because squeezed adhesive from the interface of the bonded area covered the side vias and electrodes on catheters and this prevents next electrodes patterning. Experimental results of planar and nonuniform exposure systems were evaluated in terms of resist patterning and electrode deposition after the assembly of CMUT and catheter. A requirement for different focusing points of the laser (due to planar and taper of catheter) was unable to achieve successful photoresist patterning on the catheter using the nonplanar system. LTCC side vias were used as reference points for alignment and laser exposure for nonplanar system. However, electrodes patterning on the LTCC side via and nonuniform catheter surface is a very complex and difficult process using nonuniform exposure systems. The planar exposure system, therefore, was preferred for electrodes deposition on the catheter, even on the taper. A longer exposure time is required to resist patterning on the taper than for planar surfaces of the catheter before electrode deposition. Electrodes and contact pads were electroplated with a thickness of 5 µm. Contact resistance of bonding pads after flip-chip bonding was measured using 4-wire measurement setup. Resistance of each bump measured around 2 µm although the theoretical value of a single bump was about 0.25 µm [46]. These results are considerably lower than in previous studies in literature [33]. In addition to evaluation of flip-chip bonding success, resistance between electrodes on the side via and catheter was measured with a 100% yield [46]. Figure 10 shows summary of successful packaging process flow from micromachining of the CMUT and catheter to IC mounting.

## 4. Discussion

**Figure 11:**
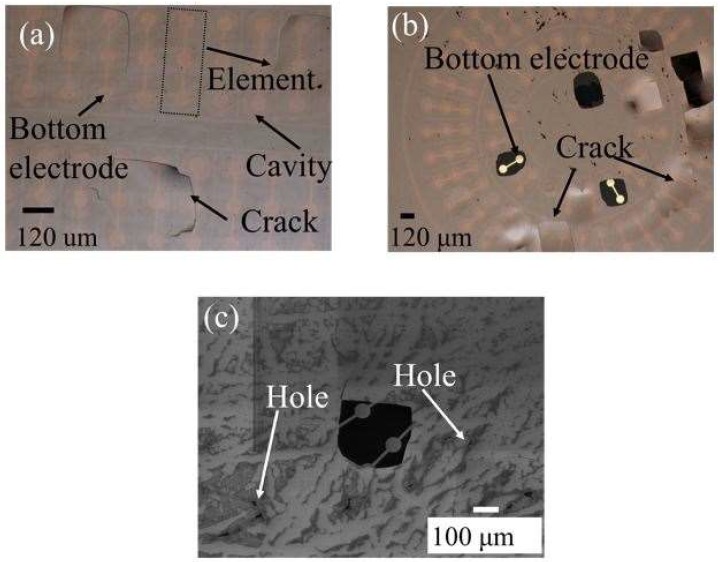
Cracks and holes observed at the CMUT membrane after handling-layer removal: (**a**) linear array; (**b**) dual ring array; and (**c**) SEM image of holes on the ring array CMUT surface after handling layer removing.

**Figure 12:**
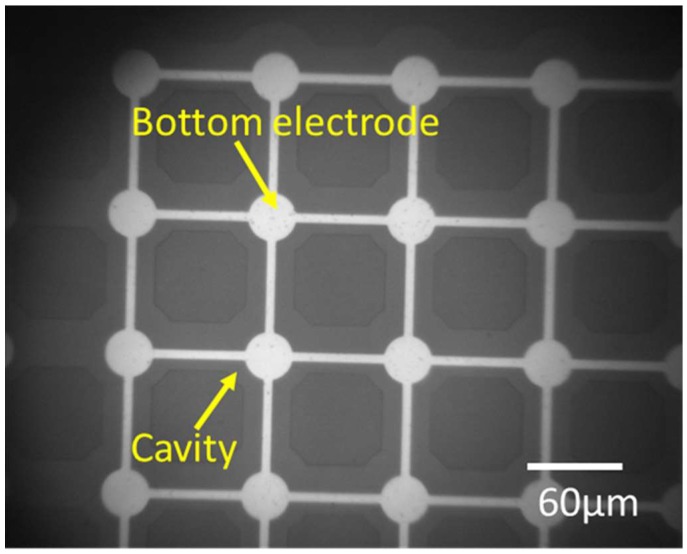
Anodic bonding result of 18 µm thick Si substrate and LTCC without cracks and holes on membrane.

In this section of paper, drawbacks, limitations, possible reasons behind undesired results of CMUT fabrication and packaging were discussed. The IR picture also revealed that no void and bubbles on the active area of the bonded surface were observed with the help of gas releasing channels. These channels were patterned between array elements and the circumference of device as shown in Figure 4d. Thus, we can say that the bonding strength was enough and voids were only visible in the gas releasing channels without significant effect on bonding strength. From the complex admittance results of three unsuccessful CMUTs, we assumed that short connections and particles on the bottom electrode that remained after the fabrication process are responsible for a parallel and a series resistor to CMUT device (capacitor), respectively. TMS results prove that the membrane deflection is upward, however, membrane displacement over the Au-Au bonded area, which was used for the electrical connection between the top and bottom surface, has a downward direction with 90 nm displacement (Figure 6c,d). Three possible reasons responsible for membrane deflection were investigated. These are gas trapped inside the cavity, the residual thermal stress on the Si surface during bonding, and TEC (thermal expansion coefficient) differences between Si and the LTCC substrate. Our previous study showed that thermal stress on the Si membrane during bonding was assumed to be a major factor behind the membrane deflection in an upward direction based on numerical analysis of thermal stress on silicon [44]. According to [44], membrane displacement due to gas trapped inside the cavity and TEC mismatch can be ignored. Considering Si membrane displacement due to high thermal stress, it can be concluded that cavities of fabricated devices were successfully sealed, however, without a vacuum due to outgassing during bonding. Previously, acoustical characteristics of the open tool LTCC-based CMUT device were shown and membrane displacement in the air was measured to be 10 times higher than in water [24]. According to our experimental results, membrane displacement in air was around 10.3 pm and the membrane displacement of our device in water should be 0.1 pm, when considering the experimental results of [24]. This displacement is very small and, thus, the output pressure of the device might not be within the range of hydrophone sensitivity for immersion measurement (acoustic). After handling layer removal, it was also observed that the silicon membrane had been removed and the membrane had collapsed to the bottom electrode in some cases, as shown in Figure 11a,b. This structure was repeatedly observed from several bonded samples. These results proved that holes and cracks at the surface of the vibration membrane made it unable to operate in the immersion medium. The Si membrane at this moment (after handling layer removing) might not be stiff enough to maintain its shape after handling layer removing. Nonlinear behavior of a Si membrane known as spring hardening can be another reason for unsuccessful device characterization due to high residual stress. Moreover, a low quality Si membrane due to overdamping in air and water can also prevent device operation. As a result, unsuccessful CMUT operation in air and water is possible due to aforementioned reasons related with the Si membrane. Because it was announced that theoretical modelling of the CMUT membrane with residual stress and cracks affects device performance (eigen frequency) [51,52,53], a thicker Si membrane can be a potential solution to eliminate cracks and holes on the membrane surface in addition to obtaining a stiffer membrane. To confirm this, Si membrane having 14 µm thickness and 80 µm diameter was used for anodic bonding with open tool LTCC. As shown in Figure 12, IR pictures of CMUT from the top side show no misalignment and cracks/holes on the membrane after anodic bonding.

**Figure 13:**
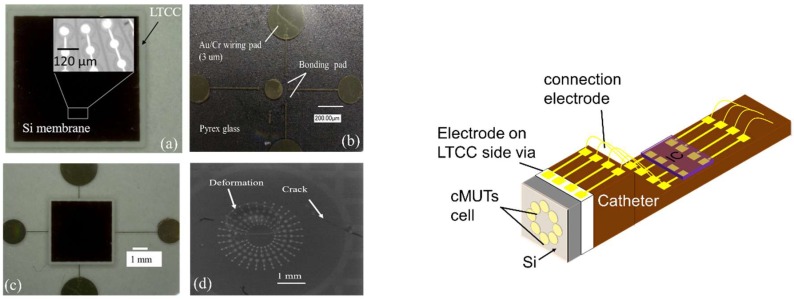
Direct integration of CMUT on dummy IC (**left**) and proposed indirect CMUT- IC connection scheme using flip-chip bonding and wire bonding (**right**).

The packaging process used in this was designed for indirect integration of CMUT with IC. Besides the advantages of CMUT packaging with LTCC, drawbacks and limitations of these new suggested methods should be considered for further improvements. From the packaging results, we can say that small size CMUT packaging is possible by using indirect connection of device and electronics rather than direct bonding. Excitation of a single CMUT array through two side vias (one is ground and other is hot electrode) might be easier than excitation of a single CMUT cell and dual ring array through multiple side vias due to a small via pitch. Moreover, it was assumed that insufficient heat flow, force, and bending of the catheter during flip-chip bonding were the main reasons behind the high contact resistivity of bump after bonding. Another micromachining method would be investigated or simple CMUT and catheter geometry would be used for electrode patterning and flip-chip bonding to gain more reliable results due to the difficulties of electrodes patterning on the nonuniform shape of the catheter. A square shaped CMUT and catheter, for example, can be a possible approach to drive a CMUT cell successfully from IC circuits patterned on a catheter. Because the surface roughness of the square shaped catheter was about the 2 µm, significantly lower than lateral side of hexagonal shape catheter (20–25 µm), the taper is no longer needed. Moreover, during the direct integration process, it is inevitable to prevent mechanical damages on a vibrating membrane of CMUT. To verify and validate mechanical damages on the active area of CMUT, CMUT was mounted on a dummy substrate (Pyrex glass) with bonding pads using flip-chip bonding by applying 25 N during 3 min. Cracks on the surface and deformed cells were observed after the bonding process (Figure 13a). LTCC side via approach for different CMUT shapes rather than a hexagon, therefore, can enable more functional and high performance CMUT fabrication and packaging. When a small size CMUT is required, we propose a packaging method where the connection between a square shaped CMUT and an IC circuit can be achieved using wire bonding and the flip-chip bonding by eliminating the taper on the catheter. This proposed packaging design is illustrated in Figure 13b.

## 5. Conclusions

In this paper, we have discussed various aspects of CMUT fabrication and packaging using LTCC substrate. Circular shaped CMUT cells with different sizes were successfully fabricated by LTCC-SOI anodic bonding. Infrared images and complex admittance plots were used to evaluate the bonding quality of dual, single ring array, and linear array CMUT. Device characteristics were investigated by obtaining equivalent circuits of devices derived from admittance plots. Our results showed that CMUT membrane size optimization does not easily achieve successful device fabrication due to via fabrication error and mechanical error of the bonding machine. It was found that a fabricated device is the only capacitor when a CMUT has a 72 µm membrane diameter. However, a resistive component was observed in the case of a CMUT with a diameter of 80 µm. Static membrane deflection at atmospheric pressure was measured to validate hermiticity of the cavity. The resonance frequency of the CMUTs with the 120 µm membrane diameters were measured at 2.88 MHz in air with a 10.3 pm displacement. Electrical and acoustical measurement of CMUTs in air and water were unsuccessful due to fabrication process related cracks and holes on the vibrating membrane. It is concluded that this caused a short connect between the top and bottom electrodes. These results indicated that the LTCC based CMUT might be suitable for air coupled applications such as gas sensing rather than immersion medium.

3D Integration of CMUT with an integrated circuit (IC) has been also investigated by using the lateral side via of LTCC. Micromachining, assembly, and electronic integration of the CMUT and catheter were presented. The LTCC side via was obtained by micromachining the CMUT into a hexagon. Connections between the CMUT and IC were achieved through electrodes patterned on a catheter that was mounted at the backside of CMUT. Electrode thickness was optimized to prevent disconnection between CMUT and IC. It was found that 5 µm electrode thickness was high enough to drive CMUT successfully. Contact resistance of flip-chip bonding was measured using a 4-wire measurement. 2 µm contact resistance was measured which is an acceptable range compared to previous studies. This indirect packaging technology might enable the integration of CMUT and integrated circuit (IC) for small sizes of ultrasonic systems.

## Figures and Tables

**Table 1:** The physical parameters of fabricated CMUT devices.

Parameters	Value
Membrane diameter (µm)	48, 50, 72, 80, 120
Membrane thickness (µm)	2.6
Cavity depth (µm)	0.4
Electrode thickness (nm)	120
Number of elements	25, 34, 54
